# Effects of *Bifidobacterium animalis* subsp. *lactis* BB-12^®^ on the lipid/lipoprotein profile and short chain fatty acids in healthy young adults: a randomized controlled trial

**DOI:** 10.1186/s12937-017-0261-6

**Published:** 2017-06-29

**Authors:** Yujin Lee, Zhaoyong Ba, Robert F. Roberts, Connie J. Rogers, Jennifer A. Fleming, Huicui Meng, Emily J. Furumoto, Penny M. Kris-Etherton

**Affiliations:** 1 Department of Nutritional Sciences, Pennsylvania State University, 110 Chandlee Laboratory, University Park, PA 16802 USA; 2 Department of Food Science, Pennsylvania State University, 206 Rodney A. Erickson Food Science Building, University Park, PA 16802 USA

**Keywords:** Probiotics, BB-12, Lipids, Lipoproteins, SCFAs, Waist circumference

## Abstract

**Background:**

Some probiotics have hypocholesterolemic effects in animal studies, which are mediated, in part, by increases in fecal short chain fatty acids (SCFAs). Clinical trials of probiotics on lipids/lipoproteins are inconsistent.

**Objective:**

We examined the effects of *Bifidobacterium animalis* subsp. *lactis* BB-12^®^ (BB-12^®^) (3.16 × 10^9^ CFUs/day) on lipids and lipoproteins and fecal excretion of SCFAs in healthy adults.

**Methods:**

In a randomized, partially blinded, 4-period, crossover study, 30 adults (11 men, 19 women) aged 18–40 years were randomly assigned to: 1) yogurt smoothie with no BB-12^®^ (YS), 2) yogurt smoothie with BB-12^®^ added pre-fermentation (PRE), 3) yogurt smoothie with BB-12^®^ added post-fermentation (POST), 4) BB-12^®^ containing capsule (CAP). We measured serum lipids/lipoproteins, glucose, insulin, C-reactive protein (CRP), and fecal SCFAs at baseline and after each treatment period.

**Results:**

Total cholesterol (TC), LDL cholesterol (LDL-C), HDL cholesterol (HDL-C), and triglycerides (TGs) did not differ after the PRE, POST, and CAP periods versus the YS or between treatments. Compared to baseline, fecal acetate was significantly increased after the YS (Δ = 211.89 ± 75.87 μg/g, *P* = 0.007) and PRE (Δ = 204.98 ± 75.70 μg/g, *P* = 0.009) periods. The percent increase in fecal acetate was significantly greater after the YS versus the POST period (52.2 ± 13.2% vs. 24.5 ± 13.2%, *P* = 0.023). Fecal total SCFAs, propionate and butyrate did not differ between treatment periods. Fecal total SCFAs were negatively associated with TC (*r* = -0.22, *P* = 0.01), LDL-C (*r* = -0.24, *P* = 0.004), age (*r* = -0.33, *P* < 0.001), and waist circumference (*r* = -0.25, *P* = 0.003).

**Conclusions:**

BB-12^®^ supplementation did not improve lipids, lipoproteins and total and individual fecal SCFAs. Fecal SCFAs were negatively associated with TC, LDL-C, age, and waist circumference.

**Trial registration:**

This trial was registered at clinicaltrials.gov as NCT01399996.

## Background

Cardiovascular disease (CVD) is the leading cause of death in the US [1]. Elevated LDL-C causes atherosclerotic CVD and, as such, it is a primary target of lipid lowering therapy [2]. Beyond pharmacotherapy, there is interest in identifying safe, inexpensive and non-pharmacological approaches for LDL-C lowering. Probiotics are defined as ‘live microorganisms that when consumed in adequate amounts confer a health benefit on the host’ by improving the composition of gut microflora [3]. Previous studies demonstrated that probiotics have beneficial effects on cancer [4, 5], diarrhea [6, 7], and irritable bowel disease [8, 9]. In addition, in our previous paper [10], we showed that probiotics have a potential anti-inflammatory effect by reducing secretion of the pro-inflammatory cytokine, TNF-α. In vitro and animal studies demonstrated that specific probiotics have hypocholesterolemic effects through possible mechanisms that include deconjugation of bile acids by bile salt hydrolase [11], production of SCFAs [12], and assimilation of cholesterol and fatty acids into the cell surface of the organism, which makes cholesterol less available for absorption into the circulation [13]. In spite of the consistent results from in vitro and animal studies, the hypocholesterolemic effects of probiotics in human studies are inconsistent. DiRienzo reviewed clinical trials that examined hypocholesterolemic effects of probiotics [14]. Some clinical trials showed that TC and LDL-C decreased after supplementation with specific probiotics [15–17] and in hyperlipidemic children [18], but other studies showed no effects [19–21]. These inconsistent results may be attributed to different strains (*Lactobacillus reuteri* NCIMB 30242, *Enterococcus faecium*, *Lactobacillus acidophilus* La5, *Bifidobacterium lactis* BB-12^®^) and doses of probiotics (4.0 x 10^6^ - 3.0 × 10^10^ CFU/g daily), delivery matrix (capsule vs. yogurt), study duration (2–60 weeks), and heterogeneous study population (healthy or with hypercholesterolemia or diabetes).

SCFAs are organic fatty acids consisting of 1 to 6 carbon atoms produced through bacterial fermentation of polysaccharide, oligosaccharide, protein, peptide, and glycoprotein precursors in the colon [22, 23]. Acetate, butyrate, and propionate are the predominant SCFAs in humans [24]. Previous studies showed that some probiotics enhance the production of SCFAs, which in turn lowered blood lipid/lipoprotein concentrations [12, 25]. The present study was designed to investigate the effects of *Bifidobacterium animalis* subsp. *lactis* BB-12^®^ on the lipid profile and fecal SCFA concentrations in healthy young adults. This is the first study to examine the effects of BB-12^®^ on the lipid/lipoprotein profile and fecal SCFA concentrations using duel delivery systems (capsule vs. yogurt) and manufacturing processes (BB-12^®^ added before vs. after the fermentation process). In addition, the association between metabolic parameters and fecal SCFAs were examined. We hypothesized that BB-12^®^ would improve the lipid/lipoprotein profile and increase fecal SCFA concentrations in healthy young adults.

## Methods

### Participants

Healthy participants (*n* = 30; 19 women, 11 men) aged 18 to 40 years were recruited. Details of the inclusion and exclusion criteria were reported previously [10]. In brief, eligibility criteria included: a body mass index (BMI) of 20–35 kg/m^2^ and delayed transit time (the amount of time between bowel movements > 24 h). Exclusion criteria included smoking, elevated blood pressure (≥140/90 mm Hg), a history of myocardial infarction, stroke, diabetes mellitus, liver disease, kidney disease, and thyroid disease. Participants taking the following supplements/medications were excluded unless they were willing to discontinue for the duration of the study: antibiotics, stool softeners, probiotics, cholesterol-lowering medications and supplements, such as psyllium, fish oil, soy lecithin, niacin, fiber, flax, phytoestrogens, and stanol/sterol supplemented foods, multivitamin, or nonsteroidal anti-inflammatory drugs. Women were excluded if they were lactating, pregnant, or planned to become pregnant during the study. The study protocol was approved by the Institutional Review Board of The Pennsylvania State University. Written informed consent was obtained from all enrolled participants at the screening visit. All the study procedures were conducted at The Pennsylvania State University Clinical Research Center (CRC). The ClinicalTrials.gov identifier is NCT01399996.

### Recruitment and screening

Participants were recruited from March 2012 - October 2013 via flyers posted at university bulletin boards, local newspaper advertisement, and email lists at Penn State University. Potential participants were contacted to conduct the initial telephone screening interview. A trained interviewer asked a list of medical and lifestyle questions. Eligible participants were scheduled for a screening appointment at the CRC. At the screening visit, anthropometric measurements (height, weight, and waist circumference), blood pressure were taken, and a fasting blood sample was collected. Details of the study recruitment and screening were reported previously [10].

### Study design and intervention

This study was a randomized, partially blinded, 4-period, balanced order, crossover design. In the partially blinded design, three treatments with yogurt smoothies were blinded to participants, and the treatment with probiotic capsule was not blinded to participants. However, all treatments were blinded to the researchers. Eligible subjects were randomly assigned to one of four treatments: 1) yogurt smoothie with no BB-12^®^ (Chr.Hansen, Milwaukee, WI) added (YS); 2) yogurt smoothie with BB-12^®^ added pre-fermentation (PRE); 3) yogurt smoothie with BB-12^®^ added post-fermentation (POST); and 4) one capsule containing BB-12^®^ (CAP). Each treatment period was 4 weeks followed by a 2-week washout period. Participants consumed either one 8-oz serving of a strawberry yogurt smoothie (220 Kcal) or one probiotic-containing capsule daily. Each smoothie/capsule contained 3.16 × 10^9^ CFUs/day of BB-12^®^. The supplemental yogurt smoothies were manufactured at the Department of Food Science at The Pennsylvania State University. Details of the manufacturing process and nutrition information for supplemental drinks were described previously [26]. Daily log sheets and weekly check-ins were completed to verify treatment compliance. In addition, participants were asked to complete 24-h-dietary recalls and physical activity records for 3 days during the last week of each treatment period. Participants agreed to maintain their weight, physical activity level, and refrain from consuming other probiotic-containing products while in the study. Weight, waist circumference, and blood pressure were measured and a fasting blood draw was taken at baseline and at the end of each period at the CRC; frozen fecal samples and completed questionnaires were returned to the CRC at the baseline and endpoint visits.

### Blood sample collection

Whole blood samples were collected by venipuncture on two consecutive days at the end of each treatment period. Participants fasted for 12 h, did not consume alcohol for 48 h, and did not take any vitamins or medication for 24 h prior to the blood draw. Whole blood was drawn into EDTA-containing tubes, centrifuged at 4 °C for 15 min. Aliquots of serum and plasma were collected and stored -80 °C until the analyses were conducted.

### Serum lipid profile

TC and TGs were measured by enzymatic analysis (Quest Diagnostics, Pittsburgh, PA; coefficient of variation [CV] < 2% for both measurements). HDL-C was measured according to the modified heparin-manganese procedure (CV <2%). LDL-C was calculated using the Friedewald equation [LDL-C = TC-(HDL-C + TG/5)].

### Serum glucose, insulin and CRP

Glucose was measured by spectrophotometry. Plasma fasting insulin was measured by immunoassay (Quest Diagnostics, Pittsburgh, PA). Serum CRP was measured by latex-enhanced immunonephelometry (Quest Diagnostics; CV < 8%).

### Measurement of short chain fatty acids

Stool samples were collected at baseline and at the end of each treatment period. Participants were provided with a fecal collection kit and asked to immediately store the fecal sample in their home freezer until they visited the CRC. Fecal samples were transported in a cooler with ice packs. The samples were stored at -80 °C prior to being aliquoted and extracted. SCFA analysis was performed according to Garcia-Villalba et al [27]. The total SCFAs represents the sum of the fecal excretion of acetate, propionate, isobutyrate, butyrate, isovalerate, valerate, and caproate.

### Questionnaires

Dietary intake was reported using 24-h dietary recalls for 3 days including 2 weekdays and one weekend day. Participants were instructed by trained study staff about how to record all foods and beverages at the baseline visit. Trained staff analyzed the 24-h dietary recalls using Food Processor SQL software (ESHA Research, Salem, OR). Physical activity was recorded using the International Physical Activity questionnaire. In brief, participants were instructed to report their activity by choosing one of nine activity categories for each 15 min interval (96 interval/day) for 3 days including 2 week days and one weekend day. Total daily physical activity was calculated by averaging the approximate metabolic equivalent of tasks (METs) of performed activities ranging from 3 to 9 over 24 h [28].

### Sample size calculation

The sample size calculation was based on a previous study [16] in which TC decreased 4.62% in the probiotic yogurt group. With α set to 0.05 and power set to 0.90, a sample size of 27 was needed for the present study. After taking into account a 10% dropout rate, the number of participants required was determined to be 30.

### Statistical analysis

Statistical analyses were performed using SAS (version 9.3; SAS Institute, Cary, NC). Normality for each variable was assessed using the univariate procedure (PROC UNIVARIATE) to check skewness. Log transformation was used if a variable was not normally distributed. All the values are presented as mean ± SEM. Baseline characteristics of participants were compared by sex using a two-sample *t*-test (PROC TTEST). *P* <0.05 was considered significant. Differences between treatment groups were assessed by analysis of variance using mixed procedure (PROC MIXED). Treatment, visit and sex were considered as fixed effects and baseline values, age, and BMI were adjusted as covariates. Participant ID was considered as a random effect. Interactions between sex and treatment group and between treatment and visit (carryover effect) were included in the model, and there were no significant interactions in the model, so they were not included in the final model. Tukey-adjusted *P* values were used for post hoc comparisons between groups. The adjusted *P* <0.05 was considered significant. Change scores were calculated by subtracting baseline values from values after each treatment. Percentage change scores were calculated by dividing change scores by baseline values, then multiplying the result by 100. The percentage change in fecal acetate was square root transformed. For glucose, insulin, and CRP, Kruskal-Wallis H test was used to compare differences among groups (PROC NPAR1WAY). For the correlation analysis, the Pearson correlation coefficient was used for normally distributed data, and the Spearman correlation coefficient was used for propionate, glucose, insulin, insulin resistance index (HOMA-IR), and CRP.

## Results

### Baseline characteristics

**Fig. 1:**
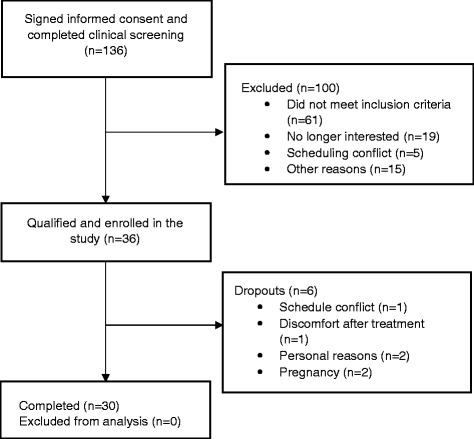
Participant recruitment flow

The study design and flow of participants were reported previously [10]. Of thirty-six participants, six participants dropped out at baseline. Data for thirty participants were analyzed (Fig. 1). Anthropometric measurements, blood pressure, metabolic parameters, physical activity index (METs) at the baseline visit are shown in Table 1. The participants (19 women and 11 men) were on average 28.2 ± 6.4 y of age with a mean BMI of 24.2 ± 2.6 kg/m^2^. BMI, waist circumference, blood pressure and biochemical markers were within normal ranges for both men and women.

**Table 1:** Baseline characteristics of participants^a^

	Men	Women	Total
*n*	11	19	30
Age, y	29.3 ± 7.0	27.6 ± 6.2	28.2 ± 6.4
BMI, kg/m^2^	23.9 ± 2.2	24.4 ± 2.8	24.2 ± 2.6
Waist circumference, cm	85.3 ± 7.5	85.0 ± 7.8	85.1 ± 7.6
Systolic blood pressure, mm Hg*	114.6 ± 7.1	102.4 ± 9.0	107 ± 10
Diastolic blood pressure, mm Hg	75.5 ± 7.2	71.9 ± 6.3	73 ± 7
TC, mg/dL	160.3 ± 29.9	168.5 ± 28.4	166 ± 29
HDL-C, mg/dL*	47.8 ± 7.1	60.6 ± 11.4	56 ± 12
LDL-C, mg/dL	92.9 ± 27.1	91.4 ± 25.2	92 ± 25
TC/HDL-C ratio	3.4 ± 0.9	2.9 ± 0.7	3.1 ± 0.8
TG/HDL-C ratio^c^	2.1 ± 0.9	1.4 ± 0.5	1.7 ± 0.7
TGs, mg/dL	97.6 ± 33.7	82.7 ± 27.8	88 ± 30
Glucose, mg/dL^b^	85.5 (82.0–90.0)	87.3 (84.0–91.0)	87.0 ± 7.4
Insulin, mU/L^b^	3.5 (1.0–6.5)	4.5 (2.5–7.0)	4.3 (2.0–7.0)
HOMA-IR	0.9 ± 0.7	1.3 ± 1.1	1.1 ± 1.0
CRP, mg/L^b^	0.5 (0.3–1.4)	0.8 (0.3–1.8)	0.7 (0.3–2.0)
Physical activity (METs) ^b^	1.7 (1.6–1.8)	1.8 (1.6–1.9)	1.7 (1.6–1.9)

^a^Values are means ± SDs; TC, total cholesterol; HDL-C, high-density lipoprotein cholesterol; LDL-C, low-density lipoprotein cholesterol; TGs, triglycerides; HOMA-IR, homeostatic model assessment-estimated insulin resistance; hs-CRP, high-sensitivity C-reactive protein; MET, metabolic equivalent of task

^b^Median; interquartile range in parentheses; *Significant difference between men and women (*P* < 0.05)

### Nutrient composition of treatments

Nutrient composition of the yogurt smoothie and BB-12^®^ containing capsule is presented in Table 2. The yogurt smoothie contained milk fat, milk solids non-fat, strawberry puree, pectin, corn syrup solids, sugar, and water. Participants consumed 8 oz. of yogurt smoothie/day that provided 220 kcals. The yogurt smoothie contained 2.5 g of fat (1.5 g of saturated fat), 10 mg of cholesterol, 90 mg of sodium, 45 g of carbohydrate (1 g of fiber and 31 g of sugar), and 7 g of protein. The nutrient composition of YS, PRE, and POST was identical except for the amount of BB-12^®^. The YS did not contain BB-12^®^ while the PRE and POST yogurt smoothies contained 3.16 × 10^9^ CFUs of BB-12^®^. The CAP contained 3.16 × 10^9^ CFUs of BB-12^®^ that provided < 2 kcals from the gelatin capsule.

**Table 2:** Nutrient composition of the yogurt smoothies^a^ (YS, PRE, and POST) and the BB-12^®^ containing capsule (CAP)

	YS	PRE/POST	CAP
Calories, kcals	220	220	<2
Total Fat, g	2.5	2.5	-
Saturated fat, g	1.5	1.5	-
Cholesterol, mg	10	10	-
Sodium, mg	90	90	-
Total Carbohydrate, g	45	45	-
Dietary Fiber, g	1	1	-
Sugar, g	31	31	-
Protein, g	7	7	-
BB-12^®^ (CFUs)	-	3.16 × 10^9^	3.16 × 10^9^

^a^Per 8 oz. of serving

### Dietary intake

Energy intake did not differ by treatment period although 8 oz. of yogurt smoothie (220 kcal/day) was added to their usual diet during YS, PRE and POST periods (Table 3). Consistently, there were no differences between the intervention periods with regard to intake of carbohydrate, protein, fat, fiber, and cholesterol. Compared with baseline, total calories carbohydrate, protein, fat, fiber and cholesterol intakes did not increase during any treatment period.

**Table 3:** Dietary intake of participants at baseline and after 4 weeks of the YS, PRE, POST, or CAPSULE treatment period^a,b^

	BL (*n* = 30)	YS (*n* = 25)	PRE (*n* = 25)	POST (*n* = 26)	CAP (*n* = 26)	*P* Value*
Total calories, kcal/d	2472 ± 152	2224 ± 164	2338 ± 161	2092 ± 165	2321 ± 162	0.43
Carbohydrate, g/d	305 ± 21	287 ± 23	291 ± 22	270 ± 23	305 ± 21	0.79
Carbohydrate, % of energy	49.3 ± 1.5	50.5 ± 1.6	50.9 ± 1.6	51.8 ± 1.6	50.6 ± 1.6	0.75
Protein, g/d	97.3 ± 6.7	94.6 ± 7.3	95.8 ± 7.2	84.4 ± 7.3	93.3 ± 7.2	0.67
Protein, % of energy	15.8 ± 0.6	17.0 ± 0.7	16.7 ± 0.7	16.2 ± 0.7	16.0 ± 0.7	0.44
Fat, g/d	96.9 ± 6.1	79.2 ± 6.7	87.0 ± 6.5	75.9 ± 6.7	90.0 ± 6.6	0.09
Fat, % of energy	35.2 ± 1.2	32.9 ± 1.3	33.2 ± 1.3	32.5 ± 1.3	34.4 ± 1.3	0.33
Fiber, g/d	21.3 ± 1.7	21.9 ± 1.8	21.4 ± 1.8	21.0 ± 1.8	24.4 ± 1.8	0.39
Cholesterol, mg/d	310 ± 25	288 ± 27	276 ± 27	283 ± 27.4	262 ± 27	0.71

^a^Values are means ± SDs; BL, baseline; YS, yogurt smoothie with no BB-12^®^; PRE, yogurt smoothie with BB-12^®^ added pre-fermentation; POST, yogurt smoothie with BB-12^®^ post-fermentation

^b^Statistical analyses were calculated using sex, treatment as between subject factor and randomization ID as the within-subject factor and age and BMI as covariates

*P values are for the main effect of treatment

### Anthropometrics and blood pressure

There were no significant differences in anthropometric variables and blood pressure between intervention periods. Compared with baseline, there were no significant changes in anthropometric variables and blood pressure after all of the treatment periods (Data not shown).

Associations between SCFAs and metabolic parameters are shown in Table 4. A negative correlation was shown between BMI and butyrate excretion (*r*_p_ = -0.18, *p* = 0.04). In addition, there was a negative correlation between waist circumference and the total SCFAs (*r*_p_ = -0.25, *p* = 0.003), acetate (*r*_p_ = -0.23, *p* = 0.006), and butyrate (*r*_p_ = -0.25, *p* = 0.003) excretion. Age was negatively correlated with the fecal excretion of total SCFAs (*r*_p_ = -0.33, *p* < 0.001), acetate (*r*_p_ = -0.24, *p* = 0.005) butyrate (*r*_p_ = -0.31, *p* < 0.001), and propionate (*r*_s_ = -0.39, *p* < 0.001)

**Table 4:** Correlations between metabolic parameters and short chain fatty acids at all time points^a^

	TC	HDL	LDL	N-HDL	TG	TC:HDL	TG:HDL	Glucose	Insulin	HOMA	CRP	Age	BMI	WC
Total SCFAs	-0.22^*^	-0.001	-0.24^*^	-0.24^*^	-0.09	-0.13	-0.05	0.12	0.16	0.16	-0.12	-0.33^**^	-0.16	-0.25^**^
Acetate	-0.18^*^	0.04	-0.24^**^	-0.22^*^	-0.03	-0.12	-0.004	0.13	0.18^*^	0.19^*^	-0.14	-0.24^**^	-0.16	-0.23^**^
Butyrate	-0.27^**^	0.01	-0.30^**^	-0.29^**^	-0.12	-0.17^*^	-0.07	0.05	0.08	0.08	-0.08	-0.31^**^	-0.18^**^	-0.25^**^
Propionate	-0.25^**^	-0.08	-0.22^**^	-0.22^*^	-0.08	-0.12	-0.04	0.17^*^	0.20^*^	0.21^*^	0.05	-0.39^**^	0.07	-0.10

^a^Pearson coefficients were used for total SCFAs, acetate, butyrate; spearman coefficients were used for propionate, glucose, insulin, HOMA-IR, and hs-CRP; TC, total cholesterol; HDL-C, high-density lipoprotein cholesterol; LDL-C, low-density lipoprotein cholesterol; N-HDL, non-high-density lipoprotein cholesterol; TGs, triglycerides; HOMA-IR, homeostatic model assessment-estimated insulin resistance; CRP, C-reactive protein; BMI, body mass index; WC, waist circumference. ^*^ *P* < 0.05, ^**^ *P* < 0.01

### Serum parameters

Compared with baseline, in response to the treatments, there were no significant changes in TC, HDL-C, non-HDL-C, TGs, TC: HDL-C, insulin, glucose, HOMA-IR, and CRP. In addition, the serum parameters did not differ by the treatment period (Table 5).

**Table 5:** Serum parameters of participants at baseline and after 4 weeks of the YS, PRE, POST, or CAPSULE treatment period^a,b^

	BL (*n* = 30)	YS (*n* = 25)	PRE (*n* = 25)	POST (*n* = 26)	CAP (*n* = 26)	*P* Value*
TC, mg/dL	165 ± 6	167 ± 6	159 ± 6	162 ± 6	162 ± 6	0.45
HDL-C, mg/dL	54.2 ± 2.0	55.9 ± 2.0	55.7 ± 2.0	54.7 ± 2.0	54.2 ± 2.0	0.48
LDL-C, mg/dL	92.3 ± 5.1	93.5 ± 5.1	93.1 ± 5.2	91.9 ± 5.2	90.3 ± 5.2	0.71
Non-HDL-C, mg/dL	110 ± 6	111 ± 6	111 ± 6	110 ± 6	108 ± 6	0.80
TGs, mg/dL	90.4 ± 6.3	88.9 ± 6.4	86.8 ± 6.4	89.9 ± 6.4	88.8 ± 6.4	0.95
TC/HDL-C ratio	3.15 ± 0.14	3.10 ± 0.15	3.13 ± 0.15	3.16 ± 0.15	3.12 ± 0.15	0.83
TG/HDL-C ratio	1.77 ± 0.14	1.68 ± 0.14	1.69 ± 0.14	1.76 ± 0.14	1.75 ± 0.14	0.85
Insulin, mU/L^c^	5.06 ± 0.88	4.57 ± 0.89	5.50 ± 0.90	5.39 ± 0.90	4.69 ± 0.90	0.99
Glucose, mg/dL^c^	86.9 ± 1.3	87.9 ± 1.3	88.2 ± 1.3	87.1 ± 1.3	87.7 ± 1.3	0.74
HOMA-IR	1.11 ± 0.20	1.00 ± 0.20	1.15 ± 0.21	1.19 ± 0.21	1.02 ± 0.21	0.63
CRP, mg/L^c^	2.07 ± 1.01	1.77 ± 1.05	1.29 ± 1.07	2.17 ± 1.07	3.77 ± 1.07	0.99

^a^Values are means ± SEMs; BL, baseline; YS, yogurt smoothie with no BB-12^®^; PRE, yogurt smoothie with BB-12^®^ added pre-fermentation; POST, yogurt smoothie with BB-12^®^ post-fermentation; TC, total cholesterol; HDL-C, high-density lipoprotein cholesterol; LDL-C, low-density lipoprotein cholesterol; TGs, triglycerides; HOMA-IR, homeostatic model assessment-estimated insulin resistance; hs-CRP, high-sensitivity C-reactive protein

^b^Statistical analyses were calculated using sex, treatment as between subject factor and randomization ID as the within-subject factor and age, BMI as covariates

^c^Statistical analyses were assessed by Kruskal-Wallis test

*P values are for the main effect of treatment

Serum TC, LDL-C, and non-HDL-C were negatively correlated with all of the fecal markers (*p* < 0.05 for all cases; Table 4).

### Fecal short chain fatty acids

**Fig. 2:**
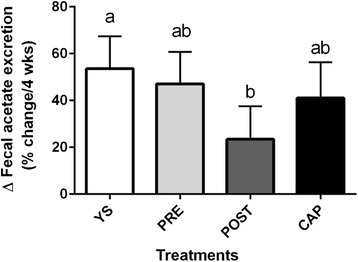
Change (%) in the excretion of fecal acetate after 4 weeks of the YS, PRE, POST, or CAPSULE treatment period. Bars are means of the percentage change. Different letters indicate significantly different values based on the mean of the square root transformed data. YS, yogurt smoothie with no BB-12^®^; PRE, yogurt smoothie with BB-12^®^ added pre-fermentation; POST, yogurt smoothie with BB-12^®^ post-fermentation; SCFAs, short chain fatty acids

Compared to baseline, fecal excretion of total SCFAs, propionate and butyrate remained unchanged after all treatment periods, whereas acetate excretion was significantly increased after the YS (Δ = 211.89 ± 75.87 μg/g, *p* = 0.007; Table 6) and PRE (Δ = 204.98 ± 75.70 μg/g, *p* = 0.009) periods. Percentage change was used to measure the magnitude of change between groups; there was a greater increase in acetate excretion after the YS period compared with the POST period. (52.2 ± 13.2% vs. 24.5 ± 13.2%, *P*_adj_ = 0.023; Fig. 2). However, there was no significant percentage change in the excretion of total SCFAs, propionate and butyrate between the treatment periods (Data not shown).

**Table 6:** Fecal SCFAs of participants at baseline and after 4 weeks of the YS, PRE, POST, or CAPSULE treatment period^a,b^

Variables	BL (*n* = 30)	YS (*n* = 25)	PRE (*n* = 25)	POST (*n* = 26)	CAP (*n* = 26)	*P*-value*
Total SCFAs, μg/g	2533 ± 208	2818 ± 219	2823 ± 216	2517 ± 220	2654 ± 219	0.66
Acetate, μg/g	701 ± 68	914 ± 71**	900 ± 70**	773 ± 71	780 ± 71	0.05
Propionate, μg/g	527 ± 47	633 ± 50	602 ± 49	529 ± 50	560 ± 50	0.32
Butyrate, μg/g	798 ± 86	889 ± 91	921 ± 90	755 ± 91	865 ± 91	0.55

^a^Values are means ± SEMs; BL, baseline; YS, yogurt smoothie with no BB-12^®^; PRE, yogurt smoothie with BB-12^®^ added pre-fermentation; POST, yogurt smoothie with BB-12^®^ post-fermentation; SCFAs, short chain fatty acids

^b^Statistical analyses were calculated using sex, treatment as between subject factor and randomization ID as the within-subject factor and age, BMI, fiber intake as covariates

*P values are for the main effect of treatment

** Values are significantly difference from baseline (*P* < 0.01)

## Discussion

The present study assessed the effect of BB-12^®^ on the lipid/lipoprotein profile and fecal SCFAs excretion. We found that daily BB-12^®^ consumption for 4 weeks did not affect lipid or lipoprotein concentrations (Table 5). The observed a lack of a BB-12^®^ effect on the lipid/lipoprotein profile agrees with a prior study in which a mixture of BB-12 and *L. acidophilus* La5 was tested [19]. In that study, 156 overweight men and women over 55 y randomly received one of the four treatments for 6 weeks: 1) probiotic yogurt plus probiotic capsules; 2) probiotic yogurt plus placebo capsules; 3) control milk plus probiotic capsules; and 4) control milk plus placebo capsules. The results showed that neither probiotic yogurt nor probiotic capsule alter the concentrations of TC, LDL-C, HDL-C or TG. In contrast, Ejtahed et al [16] found a 4.54% decrease in TC and a 7.45% decrease in LDL-C in 60 individuals with type 2 diabetes mellitus. These findings differ from what we found, however, the participant characteristics were different between the two studies. In the study by Ejtahed et al., the participants were 51 year of age, had type 2 diabetes and a mean baseline TC and LDL-C of 195 mg/dL and 117 mg/dL, respectively, whereas our participants did not have type 2 diabetes, were much younger (mean age of 28 years) and baseline TC and LDL-C were 166 mg/dL and 92 mg/dL, respectively. These findings suggest that the lipid lowering effect of probiotic may be limited to populations with borderline high or high TC and LDL-C. In addition, the lack of a BB-12^®^ effect on lipid/lipoprotein concentrations may be attributable to the probiotic strain we used because studies with different probiotic strains found different results [15, 17, 29, 30]. Specifically, studies of *Enterococcus faecium* demonstrated that TC and LDL-C concentrations decreased after consumption of the probiotic [15, 17, 30].

In the present study, there were no differences in total fecal SCFAs between the treatment periods. However, fecal acetate excretion significantly increased after the YS and PRE periods compared to baseline (Table 6). It is noteworthy that the fecal acetate excretion did not increase after the POST period compared to baseline. The amounts of BB-12^®^ in the PRE and POST were not different and the ingredients in the yogurt smoothie were identical (Table 2). In addition, dietary intake of participants did not differ by the treatment period (Table 3). We surmised further that the manufacturing process (PRE vs. POST) of the yogurt smoothie may influence the production of SCFAs. However, there is no evidence in the literature that the manufacturing process of yogurt smoothies influences the production of SCFAs. Therefore, further studies are warranted to elucidate effects of the manufacturing process of yogurt smoothies on the production of SCFAs.

The percentage change in acetate excretion was significantly higher after the YS period compared with the POST period (Fig. 2). This was an unexpected finding that acetate excretion increased after the YS period since BB-12^®^ was absent. Previous studies suggested that the production of SCFAs is influenced by prebiotics (indigestible carbohydrates) and probiotics (viable microorganisms that influence the gut microbial population) [31, 32]. In spite of the absence of probiotic in the YS, increased acetate excretion may come from ingredients in the yogurt smoothie, such as milk solids non-fat, strawberry puree, and pectin because SCFAs are produced from bacterial fermentation of polysaccharide, oligosaccharide, protein, peptide, and glycoprotein precursors [33].

In the present study, we found that the fecal excretion of SCFAs was negatively associated with blood lipids/lipoproteins (Table 4). Specifically, the levels of TC and LDL-C were negatively correlated with all of the fecal SCFAs. Our findings of the negative correlation between blood lipids/lipoproteins and SCFAs can be explained by previous in vitro and ex vivo studies that demonstrated that SCFAs suppress cholesterol synthesis by downregulating gene expression involved in intestinal cholesterol biosynthesis [34, 35]. Fushimi et al. showed that acetate influences serum cholesterol levels by decreasing hepatic 3-hydroxy-3-methylglutaryl-CoA synthase involved in cholesterol synthesis and increasing cholesterol 7α-hydroxylase involved in the conversion of cholesterol to bile acid in rats [36]. Consistent with this finding, a study by Fechner et al demonstrated that 4 weeks of lupin kernel fiber supplementation (25 g of lupin kernel fiber/d) increased fecal SCFAs excretion and decreased the levels of TC (9%) and LDL-C (12%) in 60 hypercholesterolemic adults. The fecal excretion of SCFAs was negatively correlated with waist circumference. However, this finding was in contrast to other human trial data demonstrating that the fecal concentration of SCFAs was higher in overweight and obese individuals compared with lean individuals [37, 38]. It is noteworthy that our findings are based on exploratory analyses. Therefore, further studies are necessary in order to clarify the association between obesity and fecal SCFA concentrations. Another finding in our study is that age was inversely correlated with fecal concentration of SCFAs (*r*_p_ = -0.33, *p* < 0.001). However, the effect of age on SCFAs is not fully understood. Yatsunenko et al [39] found that the human microbial population gradually decreases with increasing age, which may explain possible mechanisms for the inverse relationship between age and fecal SCFA concentrations.

There are strengths and limitations of our study. It was a randomized crossover study conducted with good compliance to daily BB-12^®^ consumption for a relatively long study period (22 weeks). This study was novel because of the different delivery systems (yogurt vs. capsule) and manufacturing processes (pre vs. post fermentation) tested to assess how these factors may change the efficacy of the probiotic. However, it is unclear why fecal acetate concentrations were increased after the YS and PRE periods, but not after the POST period. In addition, our sample size was relatively small (*n* = 30) and predominantly Caucasian, which precludes extrapolating the findings to the population at large.

In conclusion, our study demonstrated that supplementation of BB-12^®^ for 4 weeks did not improve lipids/lipoproteins. We did not observe significant differences in total fecal SCFA concentrations between treatment periods. However, consumption of the yogurt smoothie increased the fecal acetate excretion compared to baseline. In addition, fecal SCFA concentrations were inversely related to TC and LDL-C, waist circumference and age. Thus, increasing fecal SCFAs may benefit cholesterol lowering, although older age and increased waist circumference may attenuate the response. Future studies are needed to better understand the association between fecal SCFA concentrations and TC, LDL-C, age, and waist circumference in young, healthy individuals.

## Abbreviations

BB-12: *Bifidobacterium animalis* subsp. *lactis* BB-12^®^
BMI: Body mass index
CAP: BB-12^®^ containing capsule
CRC: Clinical Research Center
CRP: C-reactive protein
CVD: Cardiovascular Disease
HDL-C: HDL cholesterol
HOMA-IR: Insulin resistance index
LDL-C: LDL cholesterol
METs: Metabolic equivalent of tasks
PRE: Yogurt smoothie with BB-12^®^ added pre-fermentation
POST: Yogurt smoothie with BB-12^®^ added post-fermentation
SCFA: Short Chain Fatty Acids
TC: Total cholesterol
TGs: Triglycerides
YS: Yogurt smoothie with no BB-12^®^
